# The effect of 8-weeks of combined resistance training and chocolate milk consumption on maximal strength, muscle thickness, peak power and lean mass, untrained, university-aged males

**DOI:** 10.3389/fphys.2023.1148494

**Published:** 2023-03-15

**Authors:** Hakan Yapici, Mehmet Gülü, Fatma Hilal Yagin, Dondu Ugurlu, Ertan Comertpay, Oguz Eroglu, Melike Kocoğlu, Monira I. Aldhahi, Raci Karayigit, Sameer Badri AL-Mhanna

**Affiliations:** ^1^ Department of Sports Management, Faculty of Sport Sciences, Kirikkale University, Kirikkale, Türkiye; ^2^ Department of Biostatistics and Medical Informatics, Faculty of Medicine, Inonu University, Malatya, Türkiye; ^3^ Department of Emergency Medicine, Faculty of Medicine, Kirikkale University, Kirikkale, Türkiye; ^4^ Graduate School of Health Sciences, Kirikkale University, Kirikkale, Türkiye; ^5^ Department of Rehabilitation Sciences, College of Health and Rehabilitation Sciences, Princess Nourah bint Abdulrahman University, Riyadh, Saudi Arabia; ^6^ Department of Coaching Education, Faculty of Sport Sciences, Ankara University, Ankara, Türkiye; ^7^ Department of Physiology, School of Medical Sciences, Universiti Sains Malaysia, Kelantan, Malaysia

**Keywords:** supplementation, exercise, strength training, ultrasound, muscle hypertrophy

## Abstract

The overarching aim of this study was to investigate the combined effects of chocolate milk consumption (500 mL) with 8-week of resistance training on muscle hypertrophy, body composition, and maximal strength in untrained healthy men. A total of 22 Participants were randomly divided into two experimental groups: combined resistance training (3 sessions per week for 8 weeks) and chocolate milk consumptions (include 30 g protein) Resistance Training Chocolate Milk (RTCM) (Age: 20.9 ± 0.9 years old) and resistance training (RT) only (Age: 19.8 ± 0.7 years old). Muscle thickness (MT), using a portable ultrasound, body composition, body mass, maximal strength (one repetition maximum (1 RM), counter movement jump (CMJ) and peak power (PP) were determined at baseline and 8 weeks later. In the RTCM, finding showed a significant improvement in the outcomes compared to the RT group, besides the main effect of time (pre and post). The 1 RM total increased by 36.7% in RTCM group compared to 17.6% increased in the RT group (*p* < 0.001). Muscle thickness increased by 20.8% in the RTCM group and 9.1% in the RT group (*p* < 0.001). In the RTCM group, the PP increased by 37.8% compared to only 13.8% increase in the RT group (*p* = 0.001). The group*time interaction effect was significant for MT, 1RM, CMJ, and PP (*p* < 0.05), and it was observed that the RTCM and the 8-week resistance training protocol maximized performance. Body fat percentage (%) decreased more in the RTCM (18.9%) group than in the RT (6.7%) group (*p* = 0.002). In conclusion, chocolate milk (500 mL) with high protein content consumed in addition to resistance training provided superior gains in terms of MT, 1 RM, body composition, CMJ, and PP. The finding of the study demonstrated the positive effect of casein-based protein (chocolate milk) and resistance training on the muscle performance. Chocolate milk consumption has a more positive effect on muscle strength when combined with RT and should be considered as a suitable post-exercise nutritional supplement. Future research could be conducted with a larger number of participants of different ages and longer study durations.

## 1 Introduction

Muscle strength is one of the most important biomotor skills in promoting physical fitness, health and performance (Phillips and Winett, 2010; Sugaono and Tecchio, 2020). Resistance training (RT) is the primary way to significantly increase muscle strength and induced hypertrophy (Schoenfeld, 2010). It is well known that repeated exposure to RT has a positive effect on muscle mass and strength (Burke et al., 2001; Candow et al., 2001; Burke et al., 2003). An individuals’ resistance training history may influence their adaptive responses (Buckner et al., 2017). The magnitude of hypertrophic response is greater in untrained individuals compared to resistance trained (Ahtiainen et al., 2003). American College of Sports Medicine (ACSM) prescribes a minimum of two non-consecutive days per week for strength training, with 8–10 multi-joint exercises that stress the major muscle groups and perform two to three sets of 8–12 repetitions with good form (ACSM, 2009).

It is a consensus that RT has positive effects on many health-related mechanisms (Phillips and Winett, 2010). RT are safe and effective for various patient populations in preventing or treating health problems such as osteopathy, diabetes, and sarcopenia (Pescatello et al., 2004; Jones et al., 2009; Aronow et al., 2011; Westcott, 2012). Oppert et al. (2018) suggested that supervised resistance training for 18 weeks with additional whey protein intake (48 g/day) was superior to solely resistance training by means of strength gain. Further, a meta-analysis by Wirth et al. (2020) concluded that casein or whey protein taken whether before and after resistance training potentiate the lean body mass substantially. RT combined with additional casein protein consumption results in greater strength and muscle mass gain than RT alone (Snijders et al., 2015). It has been shown that 20 g of protein is sufficient for maximum stimulation of muscle protein synthesis (MPS) (Cuthbertson et al., 2005). Milk protein has a full profile of essential amino acids (AA) consisting of casein and whey are a muscle-building protein with adequate amounts of leucine responsible for MPS (Anthony et al., 2001; Norton and Layman, 2006; Layman et al., 2015). Anthony et al. (2000a) showed that orally administered leucine stimulated muscle protein synthesis (Anthony et al., 2000a; Anthony et al., 2000b). Milk protein contains essential amino acids, 80% casein and 20% whey protein (Phillips et al., 2009). In addition, casein protein consumption after RT is highly effective in increasing MPS compared to soy protein (Pasiakos and McClung, 2011). Whey is defined as “fast” protein and casein as “slow” protein (Boirie et al., 1997). Casein protein is slowly absorbed and may prolong high plasma-amino acid levels, thereby increasing whole-body protein conversion (Boirie et al., 1997). On the other hand, because whey protein is quickly absorbed, it gives the muscles the amino acids they need for MPS right after they eat it (Devries and Phillips, 2015). Chocolate milk also contains water, electrolytes, protein and carbohydrates. It is al-so very important in glycogen synthesis, repairing tissues and increasing performance. Milk consists of the desired 4:1 ratio of protein and carbohydrates (Ivy et al., 2003).

Studies have shown that milk protein is an effective beverage to facilitate adaptation to resistance training (Hartman et al., 2007; Josse et al., 2010). But according to one study, supplementing with 500 mL of chocolate milk daily in addition to 12 weeks of resistance exercise did not find significant increases in muscle strength and muscle fiber in both younger and older men. In addition, lower body exercise 2 days a week for 12 weeks was found to induce type II muscle fiber hypertrophy in older men (Mitchell et al., 2015). This may be due to the age of the study group. Indeed, the insufficient of hypertrophy of type II muscle fibers in older men may be due to anabolic resistance (Yang et al., 2012). In addition, the amount of protein consumed in the study may have been insufficient (Moore et al., 2015). It has been found that 40 g of high-quality protein consumed after RT in the elderly provides higher muscle protein synthesis compared to 20 g of protein (Yang et al., 2012). For this reason, it is necessary to increase muscle protein synthesis after RT and to consume more milk (protein) as an ergogenic support (Hartman et al., 2007). In addition, participants with previous RT experience and participants without training experience also provide different adaptations (Lopez et al., 2021). A review shows that when training stimuli are optimal (e.g., frequency, volume, duration), additional protein supplementation can improve muscle hypertrophy and performance (Pasiakos et al., 2015). According to the ACSM position stand study, the recommended frequency for training is 2–3 days per week (d.w-1) for beginner level, 3–4 d. w-1 for secondary education, and 4–5 d. w-1 for advanced training. For training load, 6–12 RM loads are recommended using loads corresponding to 1–12 RM, 1–2-min rest periods between sets at moderate speed (ACSM, 2009). Studies report that 8 weeks of strength training is sufficient to produce significant increases in muscle hypertrophy (Coburn et al., 2006) and muscle strength in different body parts in men and women (Dias et al., 2005). Studies in the literature report that protein supplementation is effective in increasing muscle hypertrophy. In the basis of the current literature, it has been revealed that consumption of whey protein as an additional to resistance exercise or as part of a weight loss or weight maintenance diet contributed to improve body composition parameters. However, not all studies found a significant protein effect. Research has focused on the effects of chocolate milk on recovery (Pritchett and Pritchett, 2012; Potter and Fuller, 2015). However, there has been limited re-search examining the potential changes in muscle thickness (MT), body composition, and performance of casein protein consumption in addition to resistance training. In addition, research about the efficacy of high protein chocolate milk ingestion during a resistance training program in young adults is limited. The aim of this study was to explore the effects of chocolate milk consumption (500 mL) with 8 weeks of resistance training on muscle hypertrophy, body composition, maximal strength and peak power (PP) in un-trained healthy men.

## 2 Methods

### 2.1 Participants

The study included healthy, untrained male university students 22 participants completed the exercises. G* power software (version 3.0.1) was used to calculate the sample size, with a target effect size 0.70, alpha 0.05 and power 0.80, yielding an estimated sample size of at least 19 participants for the two dependent groups (Faul et al., 2007). The mean ages of the participants in the RTCM (*n* = 11) and RT (*n* = 11) included in the study were 20.9 ± 0.9 years, 19.8 ± 0.7 years, and mean heights were 183 ± 6 cm, 178 ± 5 cm, respectively. Mean body weights were measured as RTCM: 73.0 ± 4.9, RT: 75.7 ± 3.9 kg, BMI values were determined as RTCM: 21.7 ± 1.4 kg/m^2^, RT: 23.8 ± 1.1 kg/m^2^. The exclusion criteria were as follows: Individuals without 1 year of resistance training experience, those taking performance-enhancing drugs, and those with health disabilities were excluded due to the possibility of impairing their ability to perform the physical tests. Participants were given detailed information about the potential risks and benefits of the study and signed a formulated consent form. The study was approved by the Kırıkkale University Non-Invasive Research Ethics Committee (2021.11.09). All study procedures were performed in accordance with the ethical standards outlined in the Declaration of Helsinki.2.2. Study Design.

**FIGURE 1:**
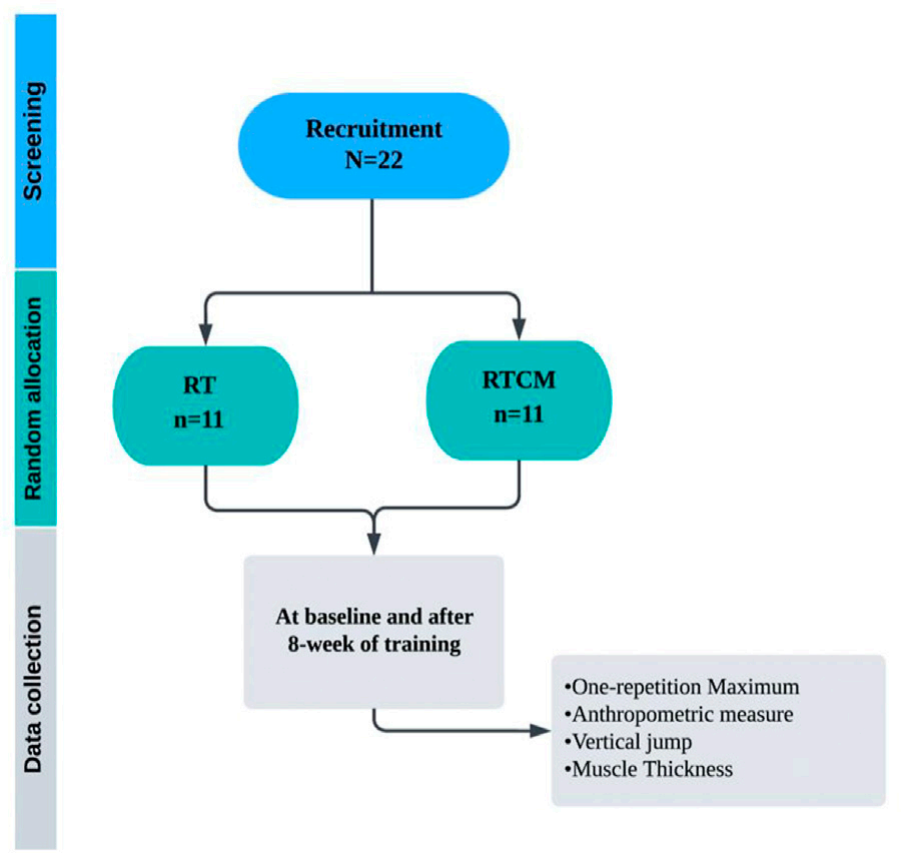
Study design.

A non-probability convenience sampling method was conducted in which healthy male volunteers who agreed to participate in the study were randomly assigned into two groups (The Resistance Training Chocolate Milk [RTCM] group, *n* = 11; The Resistance Training [RT] group, *n* = 11) (Figure 1). Randomization was based on computer-generated numbers, and revealed in the order in which participants completed baseline testing. Both intervention groups performed 8 weeks of RT for 3 days a week at the fitness center. The Resistance Training Chocolate Milk (RTCM) group consumed high-protein chocolate milk within half an hour after strength training. RT group performed only strength training at the same intensity as the RTCM group. Two weeks before the training started; the participants were given detailed information about the tests by visiting the laboratory. A week prior to the training sessions, all testing was conducted in research laboratories at Kirikkale University.

### 2.3 Training and supplementation protocol

Certified trainers checked all workouts to ensure they performed in the correct training form. After warm-up and familiarization, all the testing procedures were conducted at the same time of day and under control environmental conditions (24°C, 58%–64% relative humidity) and were performed by the same expert. After the 1RM maximum values of the participants were determined, the training loads were calculated separately for each participant. Each training session lasted approximately (55 min). Each training session consist of 8 exercises performed at volume of 3 sets of 8 repetitions with a 120-s resting interval between sets. The weekly training loads of all participants were increased by 5%.

While the RT group received only high-carbohydrate pudding in addition to the training, In the RTCM group, Energy; 191 kJ/45 kcal; saturated fat 01 g (0.1); carbohydrate 5.0 g; sugar 5.0 g; protein 6.0 g; salt 0.5 g; calcium 150 mg; (per 100 g) consumed 500 mL of chocolate milk half an hour after training. To ensure the energy balance of both groups, the participants in the RT group consumed 50 g pudding without protein (Table 1). Pudding include; Energy; 2100 kJ/502 kcal; fat 2.5 g; saturated fat 1.6 g; carbohydrate 21.7 g; sugar 4.7 g; (per 100 g).

**TABLE 1:** Total daily nutrient intakes from food diaries for all group.

	RTCM (*n* = 11)	RT (*n* = 11)
Energy (kcal)	2521 ± 322	2692 ± 381
Protein (g/day)	127.7 ± 48.1	97.3 ± 13.2
Protein (g/kg)	1.9 ± 0.7	1.6 ± 0.8
CHO (g/day)	360.2 ± 47.0	397.3 ± 47.7
CHO (g/kg)	6.6 ± 3.1	4.5 ± 1.2
Fat (g/day)	63.2 ± 10.8	79.3 ± 19.7
Fat (g/kg)	0.8 ± 0.1	0.9 ± 0.4
Calcium (mg)	1076.7 ± 97.9	1052.3 ± 87.0

CHO: carbohydrate; Kcal: kilocalories; g: Gram.

### 2.4 One-repetition maximum measurements

The participants applied two different warm-up protocols before the 1 repetition maximal values were determined. Participants cycled in the protocol for 20 min and then performed the specific warm-up (SWU protocol. They performed only the SWU before the 1RM test. In the SWU protocol, participants performed one set of 8 repetitions at approximately 50% of the estimated 1RM, followed by another set of 3 repetitions at 70% of the estimated 1RM (Brown and Weir, 2001). The test protocol was previously described by Kraemer et al. (Kraemer et al., 1991). One Repetition Maximum (1-RM) lift for Bench press (BP), Chest press (CP), Seated row (SR), Leg extension (LE), Leg curl (LC), Leg press (LP), and Squat (SQ) strength was determined, and the test consisted of two warm-up sets using three to five repetitions at 60% and 80% of the predicted 1-RM, followed by three to five subsequent trials to determine the 1-RM load. The highest mass (kg) lifted with proper form was used as the 1-RM test score.

### 2.5 Diet analysis

Participants were asked not to change their diet or restrict calories to control dietary effects. Participants recorded their food and beverage intake using a 3-day eating log be-fore and after the exercises began. Meal diaries consisted of 2 weekdays and 1 weekend day. Necessary information on how to fill in the food diary was explained to the partici-pants in detail before the research. Subsequently, the researchers noted the food diaries and clarified any misunderstandings. BeBis nutrition analysis computer application was used to examine the food diaries (BeBiS software program; Bebispro for Windows, Stuttgart, Germany; Turkish Version (Bebis 4)). The same researcher (blinded to the inter-ventions) assessed all the food diaries.

### 2.6 Peak power, vertical jump

The muscle peak power of the participants was measured in watts (W) and CMJ (cm) during vertical jump test using the Accupower 2.0 portable force platform system (Accupower 2.0, United States). Participants’ CMJ values were measured using the previously described procedure (Gülü and Akalan, 2021). The device was calibrated before the measurements were made and the measurement frequency was set to 500 Hz (Gülü and Akalan, 2021). The CMJ protocol was explained to the participants in detail. The participant was instructed to jump hands-free during the jump. Participants did a 5-min warm-up before a jump. After warming up, participants performed 3 different jumps on the strength plat-form, with 5 min’ rest between each jump. The best CMJ values and peak power values were recorded.

### 2.7 Anthropometric measurements

The height of the participants was measured without shoes, with the heels together and standing, with a height meter (Seca 217, Seca, Hamburg, Germany). Pre- and post-test Body mass was measured using a portable body analysis system accurate to 0.1 kg for the participant (Tanita Corp, Tokyo, Japan). Various methods are used to evaluate body composition. Measured components of body composition (total body fat, lean body mass, BMI, % fat) in Tanita with the BIA method. Bioimpedance analysis (BIA) which can measure several important body com-position components (Heymsfield et al., 2009). BIA measurements are based on the conduction principle of water in skeletal muscle and electrical activity in adipose tissue (Esco et al., 2011). The presence of more adipose tissue prevents the flow of electrical activity, resulting in lower electrical activity (Wagner and Heyward, 1999). By using this electric flow principle, the body fat percentage can be calculated with the BIA technique.

### 2.8 Muscle thickness measurements

MT was measured by portable ultrasonography (GE Healthcare VScan, Ultrason, General Electric Company, United States). Measurements were carried out according to the previously described procedure (Ticinesi et al., 2018). At the beginning of the measurements, each participant was asked to lie supine on a hospital bed with the knee fully extended. In addition, they were asked not to change their positions during the measurements and to maintain their resting conditions. The operator then palpated the right greater trochanter and the right intercondylar notch as landmarks of the upper and lower borders of the Vastus Lateralis muscle. Once identified, landmarks are marked on the skin with a demographic pen, and the subject is then asked to regain the resting position with the knee fully extended. The proximal-distal length between the determined points should be measured with a flexible tape measure and was accepted as the Vastus Lateralis length. MT was measured from one area: Quadriceps femoris (vastus lateralis) MT was measured as 50% between the greater trochanter and lateral epicondyle of the femur (Bridge et al., 2019). This region corresponds to where the muscle core is thickest. Participants were placed comfortably on their backs with their palms facing their bodies. A thin layer of gel was applied to the muscle area, and the ultrasound probe was placed on the area without putting pressure on the skin. The measurement was obtained by gently pressing the probe against the skin and moving it over the muscle. The MT was measured from bone to external/superficial sarcolemma. Three images were acquired from the quadriceps region and then averaged to obtain a final value. Ultrasound tests were completed at baseline and 8 weeks after training program. All tests were performed in the morning, when the participants were fasting.

### 2.9 Data analysis

In this study, the assumption of normal distribution for quantitative data was checked with the “Shapiro-Wilk” test. Quantitative data were expressed as mean and standard deviation since they showed a normal distribution. The effect of different protocols (RT and RTCM) on measurement times (Pre and Post) was determined using the Repeated Measurements two-way ANOVA test. Mauchly’s test of sphericity was used to test the homogeneity of variances and Greenhouse-Geisser correction was applied when necessary. Effect sizes for within-group, between-group and interaction effect were analyzed with partial eta-squares (η^2^p). The magnitude of differences was tested using the standardized effect size (ES) of, following the thresholds: 00.00–0.059 (small effect), 0.06–0.14 (medium effect), and ≥ 0.15 (large effect) (Cohen, 1973). The Pearson correlation coefficient was calculated to determine the relationship between muscle strength and protein intake. The following ranges were considered for the correlation coefficient sizes: 0.3–0.5 = moderate; >0.5–0.7 = large; >0.7–0.9 = very large; and >0.9 = nearly perfect (Hopkins et al., 1987). The American Psychological Association (APA) 6.0 style was used to report statistical differences (Yağin et al., 2021). Statistical data were analyzed using SPSS version 26 for Windows (IBM Corp, Armonk, NY, United States) and Python version 3.9 software. Statistical significance was set at *p* ≤ 0.05.

## 3 Results

The findings of the study showed a significant improvement in the RTCM compared to the RT group for all outcome of interest, and results were significantly higher in the post-test.

MT responses for pre and posttests following both RT and RTCM protocol are evaluated in Table 2. MT responses were different for RT and RTCM (ANOVA: group (RT vs. RTCM), F = 55.75; *p* < 0.001; η^2^ *p* = 0.74; group × time, F = 13.89; *p* < 0.001; η^2^ *p* = 0.41), moreover MT Increased with 8-week resistance training (ANOVA: time (Pre vs. Post), F = 151.94; *p* < 0.001; η^2^ *p* = 0.88).

**TABLE 2:** MT measurements analyzed using ultrasonography of the vastus lateral is muscles pre and post-test.

*n* = 22	Pre	Post	Δ	%	Time main effect	Group main effect	Interaction
					F Value	F Value	
					*p*_1_-value	*p*_2_-value	
Variable	M±SD	M±SD	TB-Tend		*η* ^*2*^_*p*_	*η* ^*2*^_*p*_	
MT (cm)							
RTCM (*n* = 11)	2.40 ± 0.3	2.90 ± 0.3	0.50 ± 0.2	20.8	F = 151.94 *p*_1_ < 0.001*η ^*2*^_*p*_ = 0.88	F = 55.75 *p*_2_ < 0.001*η ^*2*^_*p*_ = 0.74	F = 13.89 *p* = 0.001*η ^*2*^_*p*_ = 0.41
RT (*n* = 11)	2.20 ± 0.1	2.40 ± 0.1	0.13 ± 0.1	9.1			

MT: muscle thickness; M: mean values; SD: standard deviation; Δ: difference; Time Main Effect [Pre vs. Post]; Group Main Effect: [RTCM, vs. RT]; p1-value: significance test result between pre and post-test; p_2_-value: significance test result between RTCM, and RT; *statistically significant *p*-value <0.05.

In Table 3, the results anthropometric measurements of the participants’ within group, between group and interaction effect were evaluated. Weight (kg) (ANOVA: time, F = 141.95; p1 < 0.001; η^2^ *p* = 0.87; group (RT vs. RTCM), F = 5.55; p_2_ = 0.03; η^2^ *p* = 0.22; group × time, F = 13.89; *p* < 0.001; η^2^ *p* = 0.41), BMI (kg/m^2^) (ANOVA: time, F = 11.59; p1 < 0.001; η^2^ *p* = 0.85; group (RT vs. RTCM), F = 28.22; p_2_ < 0.001; η^2^ *p* = 0.58; group × time, F = 36.88; *p* < 0.001; η^2^ *p* = 0.65), and Fat (%) (ANOVA: time, F = 39.70; p1 < 0.001; η^2^ *p* = 0.66; group (RT vs. RTCM), F = 12.05; p_2_ = 0.002; η^2^ *p* = 0.38; group × time, F = 6.92; *p* = 0.02; η^2^ *p* = 0.26) decreased with 8-week resistance training and RTCM. However, Fat free (kg) (ANOVA: time, F = 63.11; p1 < 0.001; η^2^ *p* = 0.76; group (RT vs. RTCM), F = 12.24; p_2_ = 0.002; η^2^ *p* = 0.38; group × time, F = 10.22; *p* = 0.005; η^2^ *p* = 0.34) showed maximum increase with 8-week resistance training and RTCM. When the ES (η^2^p) results are examined, the larger effect between groups (RTCM vs. RT) (η^2^ *p* = 0.58) is BMI (kg/m^2^), and within-group (pre vs. post) the larger effect (η^2^ *p* = 0.87) is weight (kg) was observed.

**TABLE 3:** Anthropometric measurements baseline and after 8-week resistance training.

*n* = 22	Pre	Post	Δ	%	Time main effect	Group main effect	Interaction
					F Value	F Value	
					*p*_*1*_-value	*p*_*2*_-value	
Variable	M±SD	M±SD	T_B_-T_end_		*η* ^*2*^_*p*_	*η* ^*2*^_*p*_	
**Weight (Kg)**							
RTCM	73.0 ± 4.9	68.6 ± 4.9	4.4 ± 1.3	6.4	F = 141.95 *p*_1_ < 0.001*η ^*2*^_*p*_ = 0.87	F = 5.55 *p*_2_ = 0.03*η ^*2*^_*p*_ = 0.22	F = 49.21 *p* < 0.001*η ^*2*^_*p*_ = 0.71
RT	75.7 ± 3.9	74.6 ± 3.4	1.1 ± 0.8	1.5			
**BMI (Kg/m** ^**2**^ **)**							
RTCM	21.7 ± 1.4	20.4 ± 1.1	1.3 ± 0.5	6.4	F = 11.59 *p*_1_ < 0.001*η ^*2*^_*p*_ = 0.85	F = 28.22 *p*_2_ < 0.001*η ^*2*^_*p*_ = 0.58	F = 36.88 *p* < 0.001*η ^*2*^_*p*_ = 0.65
RT	23.8 ± 1.1	23.5 ± 0.9	0.4 ± 0.2	1.3			
**Fat (%)**							
RTCM	13.2 ± 1.5	11.1 ± 1.5	2.0 ± 0.8	18.9	F = 39.70 *p*_1_ < 0.001*η ^*2*^_*p*_ = 0.66	F = 12.05 *p*_2_ = 0.002*η ^*2*^_*p*_ = 0.38	F = 6.92 *p* = 0.02*η ^*2*^_*p*_ = 0.26
RT	16.0 ± 3.4	15.0 ± 2.6	0.8 ± 1.2	6.7			
**Fat free (Kg)**							
RTCM	9.6 ± 1.6	12.2 ± 2.9	1.9 ± 0.6	27.1	F = 63.11 *p*_1_ < 0.001 *η* ^*2*^_*p*_ = 0.76	F = 12.24 *p*_2_ = 0.002*η ^*2*^_*p*_ = 0.38	F = 10.22 *p* = 0.005*η ^*2*^_*p*_ = 0.34
RT	7.7 ± 1.6	11.4 ± 2.1	0.8 ± 1.0	48.1			

BMI: body mass index; M: mean values; SD: standard deviation; Δ: difference; Time Main Effect [Pre vs. Post]; Group Main Effect: [RTCM, vs. RT]; *p*_*1*-_value: significance test result between pre and post-test; *p*_*2*_-value: significance test result between RTCM, and RT; *statistically significant *p-*value <0.05.

In Table 4, the changes in the 1RM values of the participants were evaluated. For BP (kg) responses, time (Pre vs. Post) had a main effect and the group*time interaction effect was significant (ANOVA: time (Pre vs. Post), F = 492.10; *p*_*1*_ < 0.001; *η* ^***2***^_***p***_ = 0.96; group × time, F = 45.58; *p* < 0.001; *η* ^***2***^_***p***_ = 0.69), while RTCM and RT results were similar (ANOVA: group (RTCM vs. RT), F = 3.25; *p*_*2*_ = 0.08; *η* ^***2***^_***p***_ = 0.14). It was observed that the RTCM protocol with 8-week resistance training increased BP (kg) results. Similarly, there was no significant main effect for group in CP (kg) (ANOVA: group (RTCM vs. RT), F = 3.50; p_2_ = 0.07; *η* ^***2***^_***p***_ = 0.15), while these increased with time, and the interaction effect was significant (ANOVA: time (Pre vs. Post), F = 335.74; *p*_*1*_ < 0.001; *η* ^***2***^_***p***_ = 0.94; group × time, F = 55.41; *p* < 0.001; *η* ^***2***^_***p***_ = 0.73). CP (kg) results increased significantly with 8-week resistance training and RTCM protocol. The interaction effect was significant in SR (kg) and was highest in post-test after RTCM protocol (ANOVA: time (Pre vs. Post), F = 359.55; *p*_*1*_ < 0.001; *η* ^***2***^_***p***_ = 0.95; group × time, F = 72.81; *p* < 0.001; *η* ^***2***^_***p***_ = 0.78). The SR (kg) response were also similar between RTCM and RT protocols (ANOVA: group (RTCM vs. RT), F = 0.80; *p*_*2*_ = 0.38; *η* ^***2***^_***p***_ = 0.04).

**TABLE 4:** Baseline and post-test results of 1 RM values.

*n* = 22	Pre	Post	Δ	%	Time main effect	Group main effect	Interaction
					F Value	F Value	
					*p*_*1*_-value	*p*_*2*_-value	
Variable	M±SD	M±SD	T_B_-T_end_		*η* ^*2*^_*p*_	*η* ^*2*^_*p*_	
**BP (Kg)**							
RTCM	53.20 ± 9.0	80.50 ± 6.5	27.3 ± 4.1	51.3	F = 492.10 *p*_*1*_ < 0.001*η ^*2*^_*p*_ = 0.96	F = 3.25 *p*_*2*_ = 0.08 *η* ^*2*^_*p*_ = 0.14	F = 45.58 *p* < 0.001*η ^*2*^_*p*_ = 0.69
RT	51.40 ± 13.4	65.90 ± 13.0	14.6 ± 4.7	28.2			
**CP (Kg)**							
RTCM	65.9 ± 11.4	86.40 ± 7.8	20.5 ± 4.2	31.1	F = 335.74 *p*_*1*_ < 0.001*η ^*2*^_*p*_ = 0.94	F = 3.50 *p*_*2*_ = 0.07 *η* ^*2*^_*p*_ = 0.15	F = 55.41 *p* < 0.001*η ^*2*^_*p*_ = 0.73
RT	61.8 ± 15.8	70.5 ± 14.2	8.6 ± 3.2	14.1			
**SR (Kg)**							
RTCM	64.1 ± 6.6	90.5 ± 4.7	26.4 ± 5.5	41.2	F = 359.55 *p*_*1*_ < 0.001*η ^*2*^_*p*_ = 0.95	F = 0.80 *p*_*2*_ = 0.38 *η* ^*2*^_*p*_ = 0.04	F = 72.81 *p* < 0.001*η ^*2*^_*p*_ = 0.78
RT	69.4 ± 9.3	79.5 ± 8.5	10.0 ± 3.2	14.6			
**LE (Kg)**							
RTCM	72.3 ± 4.1	80.6 ± 7.4	17.0 ± 4.9	11.5	F = 193.40 *p*_*1*_ < 0.001*η ^*2*^_*p*_ = 0.91	F = 4.54 *p*_*2*_ = 0.04*η ^*2*^_*p*_ = 0.18	F = 14.10 *p* = 0.001*η ^*2*^_*p*_ = 0.41
RT	70.8 ± 5.4	80.6 ± 7.4	9.7 ± 4.1	13.8			
**LC (kg)**							
RTCM	60.2 ± 11.4	83.6 ± 7.1	23.4 ± 10	38.9	F = 92.15 *p*_*1*_ *η* ^*2*^_*p*_ = 0.82	F = 6.16 *p*_*2*_ = 0.02*η ^*2*^_*p*_ = 0.23	F = 17.10 *p* = 0.001*η ^*2*^_*p*_ = 0.46
RT	57.7 ± 11.6	67.0 ± 8.8	9.3 ± 3.6	16.1			
**LP (Kg)**							
RTCM	72.9 ± 15.1	94.1 ± 10.3	21.2 ± 12	29.1	F = 52.30 *p*_*1*_ < 0.001*η ^*2*^_*p*_ = 0.72	F = 14.78 *p*_*2*_ = 0.001*η ^*2*^_*p*_ = 0.42	F = 15.72 *p* = 0.001*η ^*2*^_*p*_ = 0.44
RT	64.6 ± 8.1	70.1 ± 7.3	6.2 ± 3.4	8.5			
**SQ (Kg)**							
RTCM	60.5 ± 9.6	90.5 ± 8.5	30.0 ± 5.0	49.6	F = 632.81 *p*_*1*_ < 0.001*η ^*2*^_*p*_ = 0.97	F = 0.07 *p*_*2*_ = 0.80 *η* ^*2*^_*p*_ = 0.003	F = 137.81 *p* < 0.001*η ^*2*^_*p*_ = 0.87
RT	69.1 ± 8.3	80.0 ± 7.1	10.9 ± 2.0	15.8			
**1RM total (Kg)**							
RTCM	449.1 ± 44.0	614.7 ± 30.1	165 ± 19.4	36.7	F = 1398.01 *p*_*1*_ < 0.001*η ^*2*^_*p*_ = 0.99	F = 10.92 *p*_*2*_ = 0.004*η ^*2*^_*p*_ = 0.35	F = 182.54 *p* < 0.001*η ^*2*^_*p*_ = 0.90
RT	444.8 ± 32.3	522.6 ± 319.1	78 ± 9.5	17.6			

BP: bench press; CP: chest press; SR: seated row; LE: leg extension; LC: leg curl; LP: leg press; SQ: squat; RM: repetition maximum; M: mean values; SD: standard deviation; Δ: difference; Time Main Effect [Pre vs. Post]; Group Main Effect: [RTCM, vs. RT]; *p*_*1*_-value: significance test result between pre and post-test; *p*_*2*_-value: significance test result between RTCM, and RT; *statistically significant *p*-value <0.05.

After the RTCM protocol, there was a significant increase in LE (kg) with 8-week resistance training (ANOVA: time (Pre vs. Post), F = 193.40; *p*_*1*_ < 0.001; *η* ^***2***^_***p***_ = 0.91; group, F = 4.54; *p*_*2*_ = 0.04; *η* ^*2*^_*p*_ = 0.18; group × time, F = 14.10; *p* < 0.001; *η* ^*2*^_***p***_ = 0.41) During the RTCM protocol, LC (kg) increased and this increase was higher in the post-test compared to the pretest (ANOVA: time (Pre vs. Post), F = 92.15; *p*_*1*_ < 0.001; *η* ^***2***^_***p***_ = 0.82; group (RTCM vs. RT), F = 6.16; *p*_*2*_ = 0.02; *η* ^*2*^_*p*_ = 0.23), furthermore the group*time interaction effect for LC (kg) was significant (ANOVA: group × time, F = 17.10; *p* < 0.001; *η* ^***2***^_***p***_ = 0.46). The main effect of time and the interaction effect of group*time were significant in LP (kg) (ANOVA: time (Pre vs. Post), F = 359.55; *p*_*1*_ < 0.001; *η* ^***2***^_***p***_ = 0.95; group × time, F = 72.81; *p* < 0.001; *η* ^***2***^_***p***_ = 0.78). RTCM significantly affected LP (kg) performance (ANOVA: group (RTCM vs. RT), F = 14.78; *p*_*2*_ = 0.001; *η* ^***2***^_***p***_ = 0.42) and LP (kg) was higher in RTCM compared to RT.

SQ (kg) increased after 8-week resistance training (post-test) and had a higher effect (interaction effect) with RTCM (ANOVA: time (Pre vs. Post), F = 632.81; *p*_*1*_ < 0.001; *η* ^***2***^_***p***_ = 0.97; group × time, F = 137.81; *p* < 0.001; *η* ^***2***^_***p***_ = 0.87), while there was no significantly different between RTCM and RT protocols (ANOVA: group (RTCM vs. RT), F = 0.07; *p*_*2*_ = 0.80; *η* ^***2***^_***p***_ = 0.003). Although the group main effect (RTCM or RT) was not significant for SQ (kg) performance, the group*time effect was observed to have a large effect (*η* ^***2***^_***p***_ = 0.87) and SQ (kg) showed the highest performance in the post-test after RTCM protocol. Similarly, for 1RM total (kg) results, the group*time interaction effect, time, and group main effect was significant (ANOVA: time (Pre vs. Post), F = 1398.01; *p*_*1*_ < 0.001; *η* ^***2***^_***p***_ = 0.99; group (RTCM vs. RT), F = 10.92; *p*_*2*_ = 0.004; *η* ^***2***^_***p***_ = 0.35; group × time, F = 182.54; *p* < 0.001; *η* ^***2***^_***p***_ = 0.90). Moreover, the 8-week resistance training protocol with RTCM showed the larger ES in 1RM total (kg) performance.

The group*time interaction effect was significant in CMJ (cm) performance (ANOVA: group*time, F = 14.10; *p* = 0.001; η^2^ *p* = 0.41) and increased after RTCM compared to RT (ANOVA: group (RTCM vs. RT), F = 12.27; p2 = 0.002; η^2^ *p* = 0.38). Furthermore the main effect of time was significant in CMJ (cm) (ANOVA: time (Pre vs. Post), F = 72.64; p1 < 0.001; η^2^ *p* = 0.78). Similarly, PP (watts) performance was 8-week resistance training protocol with RTCM increased significantly after *postpartum* period, that is, the group*time interaction effect was significant (ANOVA: group*time, F = 19.63; *p* < 0.001; η^2^ *p* = 0.49). There was also main effect of group and time for PP (watts) (ANOVA: time (Pre vs. Post), F = 40.71; p1 < 0.001; η^2^ *p* = 0.67; group (RTCM vs. RT), F = 13.69; p_2_ = 0.001; η^2^ *p* = 0.41) (Table 5).

**TABLE 5:** Baseline and posttest CMJ and PP results.

*n* = 22	Pre	Post	Δ	%	Time main effect	Group main effect	Interaction
					F Value	F Value	
					*p*_*1*_-value	*p*_*2*_-value	
Variable	M±SD	M±SD	T_B_-T_end_		*η* ^*2*^_*p*_	*η* ^*2*^_*p*_	
**CMJ (cm)**							
RTCM	26.80 ± 5.80	34.50 ± 3.70	7.72 ± 3.7	28.7	F = 72.64 *p*_*1*_ < 0.001*η ^*2*^_*p*_ = 0.78	F = 12.27 *p*_*2*_ = 0.002*η ^*2*^_*p*_ = 0.38	F = 14.10 *p* = 0.001*η ^*2*^_*p*_ = 0.41
RT	23.90 ± 2.40	26.90 ± 2.00	3.00 ± 1.8	12.6			
**PP (watts)**							
RTCM	2964 ± 398	4085 ± 653	1139 ± 565	37.8	F = 40.71 *p*_*1*_ < 0.001*η ^*2*^_*p*_ = 0.67	F = 13.69 *p*_*2*_ = 0.001*η ^*2*^_*p*_ = 0.41	F = 19.63 *p* < 0.001*η ^*2*^_*p*_ = 0.49
RT	2740 ± 546	2946 ± 294	205 ± 410	13.8			

CMJ: counter movement jump; PP: peak power; M: mean values; SD: standard deviation; Δ: difference; Time Main Effect [Pre vs. Post]; Group Main Effect: [RTCM, vs. RT]; *p*_*1*_-value: significance test result between pre and post-test; *p*_*2*_-value: significance test result between RTCM, and RT; *statistically significant *p*-value <0.05.

Table 6 shows the results of correlation analysis between protein intake and lean, MT, PP, and IRM results. The results showed moderate, large and very large positive correlation between PP, MT and IRM with protein intake, respectively.

**TABLE 6:** Correlation analysis results between protein, fat free, MT and PP.

Variables	Statistics	MT (cm)	PP (watts)	IRM (total)	Protein intake
**MT (cm)**	**r**	1	0.630	0.859	0.521
	*p* **-value**		0.002*	<0.001*	0.013*
**PP (watts)**	**r**		1	0.683	0.473
	*p* **-value**			<0.001*	0.026*
**IRM (total)**	**r**			1	0.705
	*p* **-value**				<0.001*
**Protein intake**	**r**				1
	*p* **-value**				

MT: muscle thickness; PP: peak power; RM: repetition maximum; *statistically significant *p*-value <0.05.

## 4 Discussion

The aim of this study was to examine the effects of chocolate milk consumption (500 mL) and 8-week of RT in young healthy men on muscle performance profile, including muscle hypertrophy, body composition, peak power and maximal strength. The main finding of this study emphasis our hypothesis which showed evidence of statistical significant and greater improvement in the muscle strength in the RTCM compared to RT group. The most improvement in 1RM, CMJ, MT, and body composition values were found in the interaction of 8-week resistance training with RTCM protocol. Findings high-lighted the effectiveness of combined RT and consumption of chocolate milk after training.

A study in parallel with our findings reported that casein protein (35 g) consumption effectively increases muscle strength and induced hypertrophy after 12 weeks of resistance training (+0.4 cm in vastus lateralis and vastus medialis) (Joy et al., 2018). In another study, in which a 12-week of resistance training and additional casein protein (27.5 g) consumption were found to provide greater strength and muscle mass gain (+11% increase in type II fiber) than resistance training without additional protein support (Snijders et al., 2015). Milk (17.5 g of protein) consumption after strength training induces greater hypertrophy in beginners compared to isoenergetic soy or carbohydrate consumption in the early stages of resistance training (*p* = 0.006) (Hartman et al., 2007). In study by Coburn et al. (Coburn et al., 2006) found that greater improvement in muscle strength and cross-sectional area (+7.31%) in those who consumed leucine (6.2 g)/whey (20 g) protein after resistance exercise compared to the group that consumed energy-compatible carbohydrate supplements. Additionally, another study reported that milk consumption promotes a positive muscle protein balance (Elliot et al., 2006). In this context, our findings support the studies in the literature, as in this study, significant improvements were found in MT in the RTCM group consuming chocolate milk compared to the RT group. In a study, the group consuming chocolate milk after resistance exercise provided significant (*p* = 0.04; ŋp2 = .08) composite muscle strength compared to the group consuming carbohydrates (Born et al., 2019). One of the main reasons for the superior gain in the RTCM group may be that it stimulated the net intake of phenylalanine and threonine, which represent net muscle protein synthesis, following resistance exercise. Studies have reported that the improvement in protein net balance of fat-free milk consumption is due to the increase in muscle protein synthesis after resistance training (Wilkinson et al., 2007). Contrary to our findings, one study reported that chocolate milk consumption after RT did not increase skeletal muscle hypertrophy (*p* = 0.52) (J. et al., 2015). Another study reported that chocolate milk or protein supplementation did not affect strength gains in the first 7 weeks of resistance training (Kuehn et al., 2015). Contrary to our findings, in studies by Kuehn et al. and Cameron et al., 2015 found that supplementing with chocolate milk did not significantly affect strength gains during the first 7 weeks of resistance training. The reason of this possibly due to insufficient training load, short training duration, or a relationship with the protein value of the milk consumed. Studies have shown that post-exercise chocolate milk consumption can provide a large intracellular signal stimulus as well as improve subsequent exercise performance (Ferguson-Stegall et al., 2011). For untrained individuals, consuming additional protein probably had no effect on lean mass and muscle strength during the first weeks of resistance training. However, as the duration, frequency, and volume of resistance training increases, protein supplementation can increase muscle hypertrophy and improve gains in muscle strength. Moreover, increased muscle mass may be associated with increased IGF-1 production with high-intensity training (Brahm et al., 1997; Goldspink, 2005).

In this study, significant increases in 1 RM as proxy of the strength were found in the RTCM group compared to the RT group, furthermore, the interaction results were observed that the 8-week resistance training protocol with chocolate milk significantly in-creased 1RM. Consistent with our findings, in study by Sharp et al. (2018) found that protein supplementation to resistance exercise resulted in improvements (+11–19%) in all groups for both deadlift and bench press compared to the 1 RM baseline. In another study, the group that consumed high-protein milk in addition to resistance exercise had superior gains compared to the control group (*p* < 0.0001) (Pourabbas et al., 2021). In untrained young men, consumption of dairy milk combined with 12 weeks of resistance exercise resulted in significant increase in type I and type II muscle fiber area (Hartman et al., 2007). In another study, chocolate milk consumption as an additional supplement to resistance training increased the muscle maximum strength (Cohen’s *d* = 0.7) (Forsyth, 2010).

Consumption of high-protein chocolate milk after exercise resulted in greater in-creases in lean mass in untrained young men compared to the RT group, these results support previous findings. A study proved that protein (46 g) consumed after RT improves body composition (Sharp et al., 2018). In the same study, protein supplement groups had a significant increase in Lean Body Mass and decrease in Fat Mass, while none of these effects were found in the control group (Sharp et al., 2018). In one study, participants consumed milk after resistance training, and a significant improvement in lean body mass was found in the milk-consuming group (Rankin et al., 2004; Pourabbas et al., 2021). In untrained young men, consuming dairy milk combined with 12 weeks of resistance exercise significantly improved lean body mass (Hartman et al., 2007). In another study, consuming chocolate milk as an additional supplement to 8 weeks of resistance training improved body fat percentage and lean body mass (Forsyth, 2010).

This study found a significant improvement in PP and CMJ values in the group that consumed high protein chocolate milk in addition to training compared to the RT group. A study by Sharp et al., shows that protein choice did not affect muscle strength outcomes, as all quality protein sources (beef protein isolate, whey protein concentrates and hydrolyzed chicken) protein showed significant improvements in maximum strength, but not significantly greater than control (Sharp et al., 2018). But only whey protein concentrates increased muscle power (Sharp et al., 2018). High protein daily milk consumption in addition to 6 weeks of resistance exercise significantly improved power compared to the control group (Pourabbas et al., 2021). Our findings support the results in the literature. The greater improvement in the group that consumed milk in addition to RT may be due to the effect of milk increasing MPS.

The results showed large level of negative correlation between Fat Free (kg) and protein measurements, while moderate, large and very large positive correlation between PP, MT and IRM with protein intake, respectively. In parallel with our findings, early intake of oral protein supplementation after RT is highly important for skeletal muscle hypertrophy in older men in response to resistance exercise (Esmarck et al., 2001). A study has found that high protein consumption is associated with muscle strength (Mangano et al., 2017). In another study, higher consumption of total, white, red and fish meat was associated with an increased index of muscle strength in young adults. Total protein intake and per-cent lean muscle mass mediated this association (Bizzozero-Peroni et al., 2022). However, another study found no association between a difference in protein intake and muscle mass in postmenopausal women (Lemieux et al., 2014). The main reason for the difference between the results of the studies may be due to the peculiarities of the methodology adopted to analyze the dietary data. In addition, the characteristics of the participant group in the research may be caused by various factors such as the amount and quality of the protein consumed, the frequency and volume of the training performed by the participants.

Although gains were obtained in MT, strength and performance with protein supplementation combined with training, yet some limitations should be acknowledged. The study was conducted among untrained male students which limit the generalizability of our finding to other subset of population because skeletal muscle responses to exercise and protein supplementation differ between trained and untrained individuals. Furthermore, findings of this study cannot be extrapolated to female due to gender disparity in the physiological profile. From physiological perspective, gender-based difference in the muscle mass and power required further investigation in future work. Another limitation of this research, that we did not assess physiological mechanism underlying the improvement in the RTCM as we did not measure the biomarkers of muscle bioenergetics system. As a another limitation that there was no control of the external activities that the participants of the sample did outside of the training or the consumption of some other ergogenic substance. Future research is required to investigate the long-term effect and the mechanisms underlying these changes in the muscle capacity and power.

## 5 Conclusion

The main findings of this study were: (i) all groups improved in body composition measurements except fat mass with improvements between groups being greater in the RTCM group; (ii) All groups had superior improvements in triceps skinfold thickness and abdominal skinfold thickness measurements in the RTCM group than in the RT group; (iii) Significant improvements were seen in all groups in MT measurements, while the most improvement was found in the RTCM group (iv) all groups showed improvements in vertical jump and peak power values, the RTCM group showed more improvement than the RT group (v) all groups were at 1 RM values showed improvement, more improvement was found in the RTCM group. In general, adaptations to strength training were found in both groups. However, in addition to strength training, consumption of high-protein chocolate milk significantly increased muscle growth compared to the RT group. Our findings can be used as a guide in training planning for mid- and long-term program design for participants with no previous resistance training experience. Future research may examine the effects on different populations of athletes, the elderly, obese individuals, and individuals with osteoporosis.

## Data Availability

The original contributions presented in the study are included in the article/supplementary material, further inquiries can be directed to the corresponding author.
